# CircRNAs in cancer metabolism: a review

**DOI:** 10.1186/s13045-019-0776-8

**Published:** 2019-09-04

**Authors:** Tao Yu, Yanfen Wang, Yu Fan, Na Fang, Tongshan Wang, Tongpeng Xu, Yongqian Shu

**Affiliations:** 1 Department of Oncology, First Affiliated Hospital of Nanjing Medical University, #300 Guangzhou Road, Nanjing, 210029 China; 2 Department of Pathology, The Affiliated Hospital of Yangzhou University, Yangzhou, 225000 China; 3 Cancer Institute, The Affiliated People’s Hospital of Jiangsu University, Zhenjiang, 212002 China

**Keywords:** Cancer metabolism, CircRNAs, Warburg effect, Lipid metabolism, Glutamine, ROS

## Abstract

Altered energy metabolism is a hallmark of tumors aiming at supplying necessary nutrients for tumorigenesis and development. These redirected metabolic pathways associated with carbohydrate, lipid and amino acid are orchestrated not only by carcinogenic proteins but by non-coding RNAs. Among them, circular RNA (circRNA), as a kind of novel identified non-coding RNAs, has become the focus of attention. Through binding with corresponding microRNAs or directly contacting proteins, circRNA plays a primarily important role in regulating cellular metabolism. Herein, we analyze the emerging findings and select circRNAs contributing to mutant glycolysis, lipogenesis and lipolysis, glutam inolysis, and oxidative respiration to deepen the understanding about the cancer metabolic regulatory network. In addition, we also discuss the possibility of circRNAs exerting their functions via exosomes and cancer stem cells. Owing to their unique structures and wide impacts, circRNAs may help reap huge fruits in developing clinical treatments targeting cancer metabolism.

## Background

**Fig. 1.**
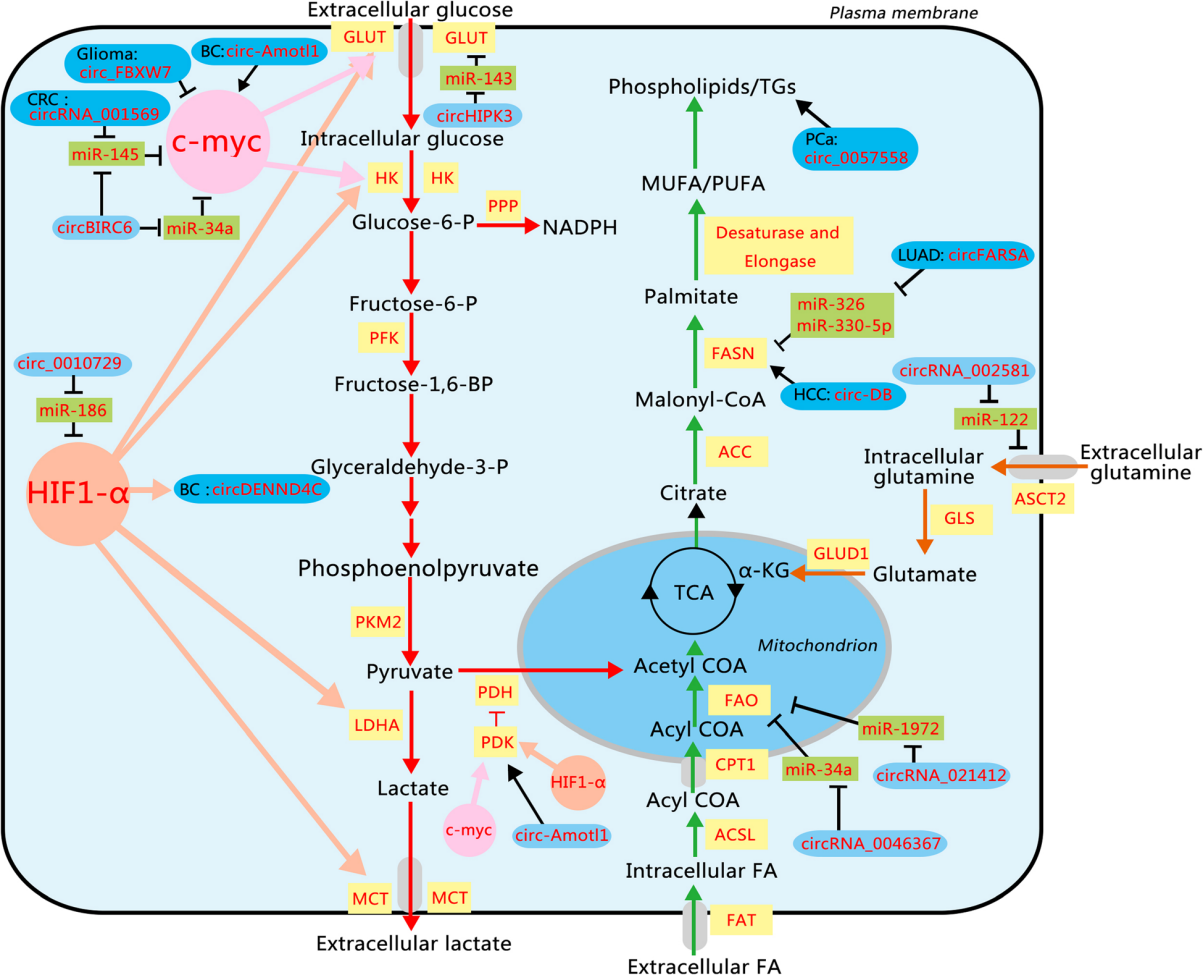
Schematic representation of altered metabolic pathways and associated circRNAs in cancer. The characteristic features include two aspects. First, it illustrates deregulated uptake of glucose and amino acids, overactive fatty acid synthesis and oxidation, and NADPH overproduction. Second, it shows how circRNAs interact with these metabolic pathways by enzymes and TFs. HIF1-α and c-myc can adjust the Warburg effect-relevant enzymes and transporters. The majority of circRNAs can antagonize miRNAs to indirectly produce effects. In addition, circ-Amotl1 and circ_FBXW7 can directly affect TFs. The circRNAs in the dark blue rounded rectangle are considered to function in cancer cells, while the circRNAs in the light blue rounded rectangle are just proved to function in normal cells. miRNAs are in the green rectangle; the components affecting metabolism are in faint yellow rectangle. The positive relationships are shown by arrows, and the negative relationships are shown by short dashes. ACC, acetyl-COA carboxylase; ACSL, acyl-CoA synthetases; ASCT2, neutral amino acid transporter2; BC, breast cancer; CPT1, carnitine palmitoyltransferase 1; CRC, colorectal cancer; FAO, fatty acids β-oxidation; FASN, fatty acid synthase; FAT, fatty acid translocase; GLS, glutaminase; GLUD1, glutamate dehydrogenase; GLUT, glutamate dehydrogenase; HCC, hepatocellular carcinoma; HK, hexokinase; LDHA, lactate dehydrogenase A; LUAD, lung adenocarcinoma; MCT. monocarboxylate transporter; PCa, prostate cancer; PDH, pyruvate dehydrogenase; PDK, pyruvate dehydrogenase kinase; PFK, 6-phosphfructa-1-kinase; PKM2, Pyruvate kinase isozymes M2; PPP, pentose phosphate pathway; α-KG, α-ketoglutarate. (Red lines, catabolic pathways; orange lines, glutamine pathways; green lines, lipid pathways)

Reprogramming of energy metabolism is a hallmark of tumors caused by genome instability [1]. Deregulated metabolism, which is widespread in tumor progression, provides an essential source for growth and division of cancer cells. Compared with normal adult tissues, carbonhydrate, lipid, and amino acid metabolisms may undergo dramatic transformational changes in tumors (Fig. 1). The recognized cancer-associated metabolic changes include deregulated uptake of glucose and amino acids, use of opportunistic modes of nutrient acquisition, use of glycolysis/tricarboxylic acid cycle (TCA), cycle intermediates for biosynthesis and nicotinamide adenine dinucleotide phosphate (NADPH) production, and increased demand for nitrogen [2]. To develop effective therapeutic strategies for cancers, it is of pivotal importance to investigate the mechanism underlying cancer abnormal metabolism.

These redirections of energy metabolism are orchestrated by both proteins and non-coding RNAs (ncRNAs). As an emerging category of regulatory molecules, ncRNA has been verified to control cancer metabolism [3]. For instance, long non-coding RNA PCGEM1 can promote glucose uptake for aerobic glycolysis in prostate cancer (PCa) cells [4]. Similarly, circular RNA (circRNA) as another type of ncRNA may also get entangled with cancer metabolism.

CircRNA is a kind of ncRNA whose structure comprises covalently closed loops without either poly-adenylated tails in 3′ ends or the cap structure at 5′ ends. Due to this extraordinary construction, circRNAs can avoid exonuclease degradation and have much longer half-life than their parental mRNAs (48 h vs. 10 h) [5]. According to this conservative property, many studies have focused their attention on the potential role of circRNAs as a promising disease biomarker. Besides, accumulating investigations have found the regulatory functions of circRNAs. They can directly regulate transcription by interacting with mRNAs or long non-coding RNAs (lncRNAs), sponging mRNAs, or RNA binding proteins (RBPs) [6, 7]. Some circRNAs can even be translated into proteins [8]. It is rational to hypothesize that circRNAs may regulate cancer metabolism through sponging miRNAs or other targets. Hence, in this article, we collect putative circRNAs affecting metabolism and divide them into three groups associated with carbohydrate, lipid, and amino acid respectively. The aim of this review is to gain insights into the relationship between circRNAs and cancer metabolism and provide a better theoretical basis for the clinical diagnosis and treatment of cancers.

## Main text

### circRNAs in glucose metabolism

The modification of glucose metabolism is part and parcel of the most distinct differentiation in cancer. Normally, cells consume glucose to generate ATP through oxidative phosphorylation (OXPHOS) under the aerobic condition and channel into glycolysis only under the anoxic condition. However, cancer cells prefer glycolysis even in the normoxic environment. This phenomenon is known as “the Warburg effect” as Otto Warburg reported this phenomenon in the 1920s [9]. The Warburg effect is instrumental in malignancy. First, the change from OXPHOS to glycolysis relieves oxidative stress injury caused by mitochondria [10]. Second, excess lactic acid produced by the Warburg effect helps avoid immune surveillance. Besides, it is worth noting that recent profiles have proved that the Warburg effect does not account for the main energy supply due to the inefficiency of glycolysis in ATP synthesis. So it is hypothesized that the Warburg effect consumes a myriad of glucose to either create a hyperacid microenvironment or just starve normal cells in the vicinity [11]. The produced lactate can also stimulate vascularization via HIF1-α [12]. Specifically, the Warburg effect is triggered by abnormal expression of glucose transporters, metabolic enzymes, and oncogenes. Several important signaling pathways also participate in the regulation of this process. Hereinafter, we illustrate the effects of circRNAs on aerobic glycolysis of all these levels (Table 1).

**Table 1:** circRNAs in glycolysis

circRNA	Mediator miRNA	Target component	Effect on glycolysis	Refs	Cancer type
circHIPK3	miR-124	GLUT2	Up	[13]	–
circ-Amotl1	–	PDK1, AKT1, c-myc, STAT3	Up	[14–16]	Breast cancer
circDENND4C	–	HIF-1α	Up	[17]	Breast cancer
circ_0010729	miR-186	HIF-1α	Up	[18]	–
circRNA_001569	miR-145	C-myc	Up	[18]	Colorectal cancer
circBIRC6	miR-145, miR-34a	C-myc	Up	[19]	–
circ-FBXW7	–	C-myc	Down	[20]	Glioma
circNRIP1	miR149-5p	AKT1	Up	[21]	Gastric cancer
circ-ZNF609	miR-150-5p	AKT3	Up	[22]	–
circRNA_103801	miR-370, miR-877	PI3K/AKT, HIF	Up	[23]	Osteosarcoma
circRNA_100290	miR-29, miR-516b	CDK6, RAS, Wnt/β-catenin	Up	[24, 25]	Oral squamous cell carcinoma, Colorectal cancer
circRNA-MYLK	miR-29a	RAS	Up	[26, 27]	Bladder cancer
circ-ITCH	miR-22-3p	CBL	Down	[28]	Papillary thyroid cancer

### circRNAs play a vital role in glycolysis by regulating transporters and enzymes

As the carbohydrate metabolism is a major energy-providing pathway, a group of metabolic enzymes work together to maintain successful running of the process. Firstly, the glucose transporter (GLUT) takes charge of the import of glucide. To meet the excessive requirements of glucose, GLUTs ranging from GLUT1 to GLUT4 are all overexpressed in tumor cells. Then, three speed-limiting enzymes including hexokinase (HK), 6-phosphfructa-1-kinase (PFK), and pyruvate kinase (PK) work together to transform glucose into pyruvate. Lactate dehydrogenase A (LDHA), which executes the final step of aerobic glycolysis, can catalyze pyruvate into lactate. Finally, the monocarboxylate transporter (MCT) releases lactate into the extracellular matrix. In addition, pyruvate dehydrogenase kinase (PDK) blocks the traditional OXPHOS by countering pyruvate dehydrogenase (PDH) that catalyzes pyruvate into acetyl-COA [29]. PDK1 is a component of the PI3K/AKT pathway to affect cell growth, differentiation, and survival [30].

Some circRNAs can affect these enzymes. Some experimental studies reported that silencing of circHIPK3, which is abundant in pancreatic islets, decreased Slc2a2 expression that encodes GLUT2 [13]. CircHIPK3 could sponge miR-124, which represses the expression of several enzymes and transporters of glycolysis [31, 32]. Another circRNA circ-Amotl1 was reported to be able to physically bind to PDK1 and AKT1 and translocate them into the nucleus in order to antagonize apoptosis [14]. These circRNAs are likely to induce the Warburg effect in tumor tissues.

### circRNAs affect glycolysis by regulating transcription factors

**Fig. 2:**
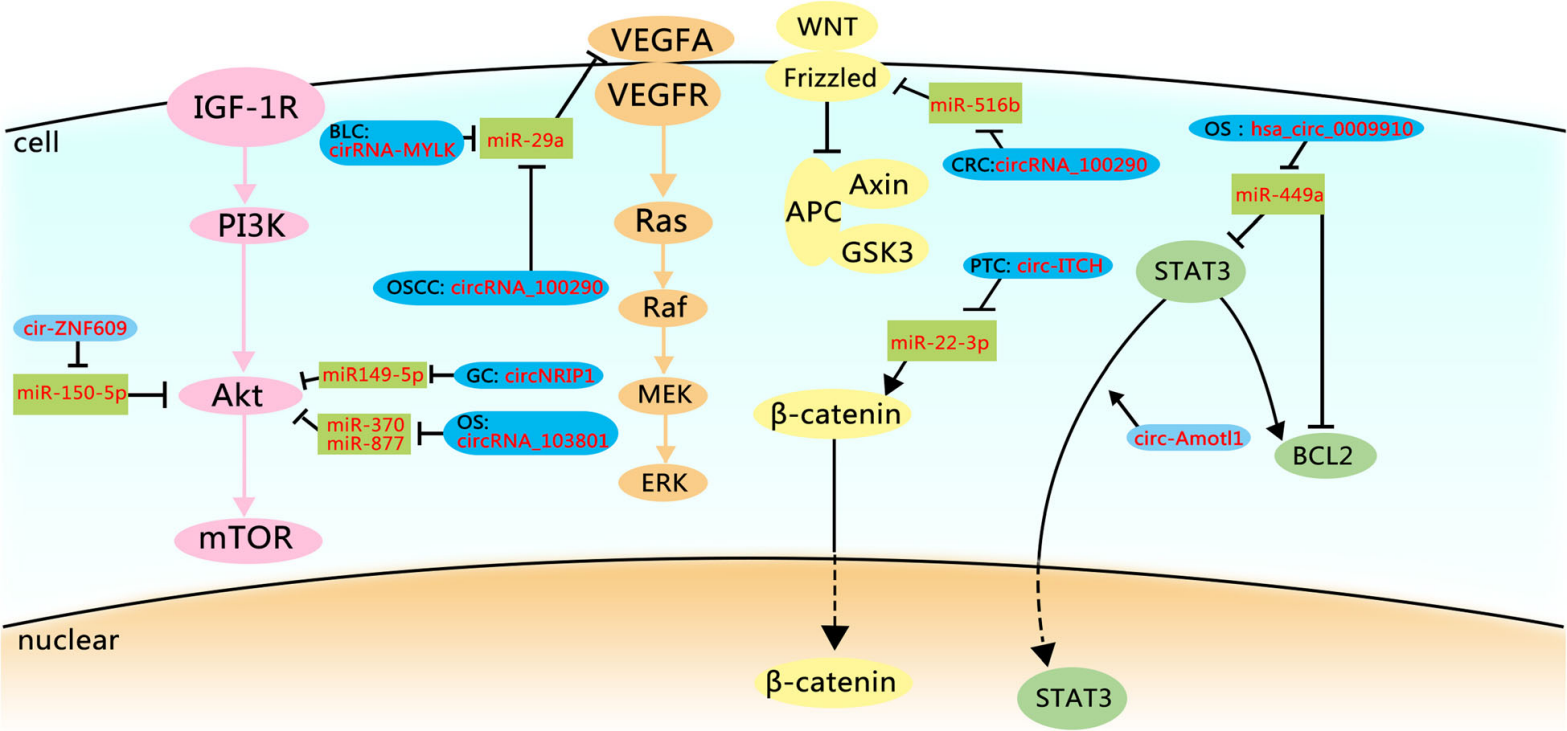
circRNAs and the Warburg effect associated signaling pathways. This figure indicates the effects of circRNAs on the Warburg effect-associated signaling pathways. The PI3K/Akt pathway promotes GLUT1, HK2, and PFK2. The alteration of RAS pathway upregulates GLUT1 and glycolytic enzymes. The STAT3 pathway gives rise to HK2. And the Wnt/β-catenin pathway upregulates the transcription of MCT1 and PDK1. In these four pathways, most circRNAs work as ceRNAs to affect the Warburg effect. The circRNAs in the dark blue rounded rectangle are considered to function in cancer cells, while the circRNAs in the light blue rounded rectangle are just proved to function in normal cells. miRNAs are in the green rectangle; the components of the PI3K/Akt pathway are in the pink circle; the components of the RAS pathway are in the orange circle; the components of the STAT3 pathway are in the green circle; the components of the Wnt/β-catenin pathway are in the yellow circle. The positive relationships are shown by arrows, and the negative relationships are shown by short dashes. BLC, bladder cancer; CRC, colorectal cancer; GC, gastric cancer; OS, osteosarcoma; OSCC, oral squamous cell carcinoma ; PTC, papillary thyroid cancer

Metabolic changes in cancer are viewed as a subsequent step of the molecular transformation process. Distinctive transcription factors (TFs) also affect the Warburg effect. As a member of ncRNAs, circRNA regulates the mutant expression of TFs. So we pay attention to the selected TFs to elucidate the relationships between circRNAs and TFs (Fig. 2).

### HIF-1

There is a tight association between hypoxia and the Warburg effect [33]. Solid tumors encountering hypoxia have to adopt glycolysis instead of oxidative metabolism. In response to the hypoxic condition, HIF-1 contributes to the overexpression of a series of carcinogenic genes. It was found that the lactate production rate increased proportionally with the level of HIF-1α, for it facilitates the transcription of genes related to GLUTs and glycolytic enzymes including HK, PKM, and MCT4 [11]. HIF-1 also enhanced the PDK1 function [34].

Because neovascularization frequently occurs at the periphery of tumors rather than within tumors, larger cancer tumors incline to suffer more severe hypoxia. Therefore, the tumor size and proliferation rate can often represent the extent of hypoxia damage. CircDENND4C, which was found to be upregulated by HIF-1α in breast cancer cells, aggressively promotes cell proliferation in a hypoxic environment [17]. Knowing that a larger mass consumes more energy and bears more extreme hypoxia, circDENND4C is supposed to enhance the Warburg effect. In human umbilical vascular endothelial cells (HUVECs), circ_0010729 coexists with HIF-1, especially in hypoxia-induced HUVECs, where circ_0010729 overexpressed and elevated the HIF-1α expression by sponging miR-186 [35]. With the support of thick vessels, more glucose is brought inside tumors. So circ_0010729 may also facilitate glycolysis by enhancing the progress of vascular endothelial cells.

### C-myc

c-myc, one of the most important oncogenic TFs, can also activate the Warburg effect. Ectopic c-myc can collaborate with HIF-1 to elevate the level of the glycolytic components GLUT1, HK2, and PDK1 [34, 36].

In bladder cancer, miR-145 could alleviate the Warburg effect by silencing c-myc [37]. To explain detailedly, polypyrimidine tract-binding protein1 (PTBP1) as the downstream of c-myc induces the up-regulation of PKM2 instead of PKM1. Meanwhile, miR-145 can counteract the function of the c-myc/PTBP1 axis to reduce the PKM2/PKM1 ratio [38]. Two circRNAs interplay with miR-145. One is circRNA_001569 sponging miR-145 in colorectal cancer, and the other is circBIRC6 [18, 19]. circBIRC6 also targets miR-34a, another miRNA repressing c-myc [19]. The above-mentioned Circ-Amotl1 can also assist c-myc in nuclear translocation in order to prevent the degradation of c-myc [15]. Besides c-myc, circ-Amotl1 could link with classic oncogenic proteins such as AKT and STAT3. Consequently, it is highly feasible that circ-Amotl1 enhances aerobic glycolysis to facilitate tumor development without working as a competing endogenous RNA (ceRNA). Intriguingly, circ-FBXW7 could encode protein FBXW7-185aa which was capable to competitively interact with the de-ubiquitinating enzyme USP28 [20]. So FBXW7-185aa could indirectly degrade c-myc. Circ-FBXW7 probably ameliorates the Warburg effect in this way.

### circRNAs affect glycolysis by regulating signaling pathways

Effective TFs work together to form a regulatory network that affects the Warburg effect. The network consists of different signaling pathways in which diverse circRNAs are involved. So reviewing the representative signaling pathways and associated circRNAs can help understand this metabolic mechanism more comprehensively.

### PI3K/Akt

The PI3K/Akt pathway plays a crucial role in motivating glucose uptake and glycogen synthesis. Akt can encourage HK2 and PFK2 to stimulate glycolysis [39]. MTOR complex1, as the downstream of Akt, can phosphorylate elf4E-binding proteins and support HIF-1α to promote GLUT1, HK2, and PFK2 [40, 41].

First of all, in gastric cancer, circNRIP1 could upregulate glucose uptake and induce lactification via sponging the miR149-5p that targets AKT1 [21]. Second, it is possible that miR-150 directly depresses the Warburg effect. Downregulation of miR-150 reinforced the resistance of ovarian cancer cells to pertuzumab by increasing p-Akt [22]. And in osteosarcoma, miR-150 might repress glycolysis by targeting the 3′-UTR of GLUT1 [42]. In Hirschsprung disease, the level of AKT3 scaled up with that of circ-ZNF609, while miR-150-5p expression was inversely associated with circ-ZNF609 and AKT3 [43]. Some experimental studies found circ-ZNF609 could harbor the target sites of miR-150-5p. So circ-ZNF609 might promote AKT3 by targeting miR-150-5p to improve the Warburg effect. More research is required to confirm its role in cancer metabolism. In osteosarcoma, circRNA_103801 was found to be associated with the HIF-1 and PI3K/AKT pathway by binding with miR-370 and miR-877 as predicted by bioinformatics tools [23]. In ovarian cancer, miR-29b targeted AKT2 and AKT3 to moderate the Warburg effect [44]. And in oral squamous cell carcinoma, circRNA_100290 sponged miR-29 family to downregulate CDK6 [24]. Similarly, miR-124 could target AKT1 and AKT2 to decrease GLUT1 and HK2 to relieve the Warburg effect in non-small cell lung cancer cells [31]. And in vascular smooth muscle cells, circWDR77 has been verified to sponge miR-124 [45].

### RAS

KRAS mutations occur in a variety of cancers, especially in colorectal cancer, non-small cell lung cancer, and PCa. The alteration of KRAS upregulated GLUT1 and glycolytic enzymes to facilitate the Warburg effect [46]. In bladder cancer, ectopic circRNA-MYLK increased the expression of RAS and the downstream Raf/MEK/ERK [26]. CircRNA-MYLK can activate the vascular endothelial growth factor A to elevate the glucose brought by blood as a ceRNA for miR-29a. Additionally, depending on the ceRNA network, both lncRNA H19 and circRNA-MYLK were the ceRNA of miR-29a-3p in breast cancer [27]. And H19 could activate PKM2 and PKM2 polymers in hepatocarcinogenesis, with the upregulation of c-myc and HRAS [47]. Deductively, circRNA-MYLK might sponge miR-29a to discharge more H19 to increase PKM2. Similarly, circRNA_100290 sponged the entire miR-29 family, which is perhaps helpful for the expression of RAS.

### STAT3

The STAT3 pathway gives rise to the inflammatory environment in favor of cancerogenesis. Experiments in breast cancer cells have demonstrated that STAT3 could directly upregulate the expression of HK2 [48]. The aforementioned circ-Amotl1 could communicate with STAT3 to markedly increase cell proliferation [16]. By increasing the expression of STAT3 and inducing STAT3 nuclear distribution, circ-Amotl1 enhanced STAT3 expression. In osteosarcoma, hsa_circ_0009910 indirectly facilitated STAT3 with miR-449a as a mediator [49]. Circ_0009910 could sponge miR-449a which antagonized IL6R. IL6R increased BCL-2 and STAT3 in hepatocellular carcinoma (HCC). The deregulated circ_0009910 could cut down the levels of p-STAT3 and Bcl-2 [50].

### Wnt/β-catenin

The Wnt/β-catenin pathway can direct the Warburg effect by upregulating the transcription of MCT1 and PDK1 [12]. Mutant Wnt signaling can activate β-catenin leading to the overactive response of the downstream genes. Furthermore, c-myc has been ascertained as a Wnt/β-catenin target [28]. In papillary thyroid cancer, circ-ITCH could sponge miR-22-3p to upregulate CBL, an E3 ligase of nuclear β-catenin [51]. CBL, which is detrimental to tumor growth and development, could degrade β-catenin. So circ-ITCH might suppress the Warburg effect by impairing the activation of the Wnt/β-catenin pathway. Additionally, circRNA_102171, circ_0067934, and circRNA_100290 were in favor of the Wnt/β-catenin pathway by targeting CTNNBIP1, miR-1324, and miR-516b, respectively [25, 52, 53], while circ_0006427 inactivated the Wnt/β-catenin pathway by targeting miR-6783–3p [54].

### circRNAs in lipid metabolism

Exacerbated lipid production is another hallmark of tumor metabolism, for cancer cell growth requires fatty acids (FAs) to replicate cellular membranes. Endogenous fatty acid synthesis (FAS) is triggered in various tumors [55]. Acetyl-COA can be converted from citrate catalyzed by ATP citrate lyase. The first step of FA biosynthesis is the transformation of cytosolic acetyl-COA to malonyl-COA via acetyl-COA carboxylase. Then, fatty acid synthase (FASN) catalyses malonyl-COA into long FA chains [2]. In contrast, altered lipid mobilization also has relevance with tumor progression. For example, increased lipolysis leads to a wasting syndrome known as cancer cachexia, which is characterized by acute fat loss [56]. Tumor cells can acquire FAs through lipolysis to perform FA β-oxidation (FAO) which provides energy for tumor proliferation. Due to the Warburg effect, less acetyl-COA is generated by glucose, while FAO can comprise this deficiency. Moreover, lipophagy, meaning autophagic degradation of lipid, is another pathway of lipid utilization [57]. Recent studies indicate that lipophagy is a well-conserved mechanism of lipid degradation. Lysosomes can digest lipid droplets with the help of phagophore to reproduce free FAs [58].

An inverse correlation between circRNA_0046367 expression and triglyceride (TG) level in HepG2 cell culture and liver tissues has been reported [59]. CircRNA_0046367 could sponge miR-34a to protect the peroxisome proliferator-activated receptor (PPAR) α from transcriptional repression. PPARα activates CPT2 and ACBD3 to degrade lipids [60]. So circRNA_0046367 might promote tumor progression partly through upregulating FAO. CircGFRA1 attracted miR-34a in triple negative breast cancer as a ceRNA to release the restrain of GFRA1, which can induce autophagy [61, 62]. More studies are required to confirm whether circGFRA1 concomitant with GFRA1 can promote lipophagy in cancer metabolism. On the contrary, circ-DB released by adipocyte exosomes also sponged miR-34 but appeared to raise the body fat ratio in HCC patients [63]. HCC patients with the higher body fat ratio incline to have more rapid tumor progression and poorer prognosis. In this circumstance, circ-DB seems to facilitate HCC progression via FAS. However, this mechanism is paradoxical to circRNA_0046367 whose target is miR-34a as well. Considering that the expression of miR-34a is increased in obesity causing the fat browning and the weight loss, the difference of circ-DB function may be caused by lipid in exosomes [64]. But the authentic mechanism remains elusive. After high fat stimulation, circRNA_021412 was downregulated in HepG2 cells causing reactivation of miR-1972 [65]. Increased miR-1972 reduced the level of LPIN1, which can coordinate with PPARα to recruit long-chain acyl-CoA synthetases. The inhibition of LPIN1 blocks FAO resulting in hepatic steatosis. The circRNA_021412/miR-1972/LPIN1 axis may also be involved in tumor progression. The expression of circ_0057558 was found to be positively associated with the level of TG in PCa [66]. PCa is a hormone-dependent cancer influenced by lipid metabolism abnormalities [67]. So circ_0057558 might regulate the level of lipid to promote PCa progression. CircFARSA, which was found to be highly expressed in lung adenocarcinoma, is supposed to interact with FASN through miR-330-5p and miR-326 in accordance with the in silico analysis [68]. And in laryngeal squamous cell carcinoma, hsa_circ_0033988 was lowly expressed and associated with FA degradation according to ceRNA network analysis and functional annotation analysis [69]. These results computed by special algorithms have yet to be further validated by experiments in vitro and in vivo (Table 2).

**Table 2:** circRNAs in lipid metabolism

circRNA	Mediator miRNA	Target component	Effect on lipid metabolism	Refs	Cancer type
circRNA_0046367	miR-34a	PPARα	Promoting FAO	[59]	–
circGFRA1	miR-34a	GFRA1	Promoting lipophagy	[62]	Triple negative breast cancer
circ-DB	miR-34a	–	Promoting FAS	[63]	HCC
circRNA_021412	miR-1972	LPIN1	Promoting FAO	[65]	–
circ_0057558	–	–	Promoting TG production	[66]	PCa
circFARSA	miR-330-5p, miR-326	FASN	Promoting FAS	[68]	Lung adenocarcinoma
circ_0033988	–	–	Promoting FA degradation	[69]	Laryngeal squamous cell carcinoma

### circRNAs in amino acid metabolism

Since the 1950s, scientists have found that tumor cells consume much more glutamine than any other amino acid. Glutamine can be converted to glutamate catalyzed by glutaminase. Subsequently, glutamate can provide nitrogen for purine and pyrimidine biosynthesis, or participate in the TCA cycle by transforming to α-ketoglutarate. Glutathione, an important cellular antioxidant, also comes from glutamate. Thus, glutamine plays an essential role in tumor progression.

Despite the deficiency of direct evidence, scientists have investigated the impact of circRNAs on glutamine metabolism by comprehensive analysis (Table 3). In glioblastoma, functional analysis predicted that circRNAs may participate in glutamergic synapse and calcium signaling [70]. Computational analysis discovered the circRNA_002581/miR-122/Slc1a5 axis in non-alcoholic steatohepatitis [70]. Knowing that Slc1a5 is a kind of glutamine transporter, biological research is needed to evaluate the relationship between circRNA_002581 and glutamine metabolism [71].

**Table 3:** circRNAs in glutaminolysis

circRNA	Mediator miRNA	Target component	Refs	Cancer type
circRNA_002581	miR-122	Slc1a5	[70]	–

### circRNAs in oxidative respiration

Owing to the electron transport flux accumulated by proliferating cells and hypoxia in the tumor microenvironment, the constant generation of reactive oxygen species (ROS) is another attribute of cancer. Overexpressed ROS can develop into oxidative stress, eventually resulting in oncogene-induced cellular senescence. Although ROS overproduction is harmful for cell growth, a moderate amount of ROS is beneficial for the maintenance of a tumorigenic condition [72]. The tolerable level of ROS not only activates HIF1-α and NRF2 but inhibits protein phosphatases such as PTEN [2]. So reduction-oxidation (redox) homeostasis is a crucial component of cancer metabolism. Some studies demonstrated that several circRNAs were differentially expressed in substantia nigra and corpus striatum of NRF2 knock-out mice compared with the control group [73]. Increases CircNCX1 in response to ROS in cardiomyocytes could sponge miR-133a-3p to release the pro-apoptotic gene CDIP1 [74]. These circRNAs implicated with ROS may also regulate the redox equilibrium in tumor cells [Table 4].

**Table 4:** circRNAs in oxidative respiration

circRNA	Mediator miRNA	Target component	Effects	Refs	Cancer type
circNCX1	miR-133a-3p	CDIP1	Regulating redox	[74]	–
circ_0062019	–	SLC19A1	Promoting folate transport	[66]	PCa

The folate cycle plays a dominant role in the generation of NADPH, which effectively antagonizes ROS. Recent studies point out that folate is required for HCC and PCa cells growth [75, 76]. In PCa, circ_0062019 and its host gene SLC19A1 were significantly upregulated [66]. SLC19A1 can encode a membrane protein to transport folate. This result suggests that circ_0062019 may act as a booster to further PCa proliferation by the folate cycle.

## Inductive analysis

Major regulatory mechanisms of circRNAs can be divided into three aspects. Firstly, circRNAs with miRNA response elements can serve as ceRNAs to sponge miRNAs [6]. As circRNAs lack poly (A) tails and 5′ ends, it is difficult for them to get degraded. Thereby, a small portion of circRNAs can repress a large number of miRNAs [77]. Secondly, it can interact with RNA polymerase II or splicing factors to regulate gene transcription [78]. Finally, they can combine with RBPs to compete with RBP substrates. According to the up-to-date records, we find that most circRNAs regulate cancer metabolism through sponging miRNAs. In addition, circ-FBXW7 as a substitution of its linear counterpart attracted USP28. Circ-Amotl1 induced the nuclear translocation of PDK1, AKT1, and c-mcy. These special mechanisms broaden the horizon on the research of circRNAs and might bridge to novel regulatory systems.

As a core hallmark of cancer, altered energy metabolism has its particularity and university. On the one hand, metabolism consists of distinctive chemicals, transporters, and enzymes, running for supplying necessary nutrients. On the other hand, deregulating cellular energy metabolism is also supported by TFs and ncRNAs that are implicated in other core hallmarks of cancer, such as proliferation and metastasis. To some extent, altered energy metabolism is simply another profile of oncogene programming. Therefore, for one thing, we gather the circRNAs that directly regulate enzymes, like circHIPK3 that affects GLUT1. For another, we record the circRNAs related to the TFs and pathways which assist in the formation of abnormal metabolic process, such as circ-Amotl1 and circRNA_100290. The results observably ascertain that circRNAs are of importance in regulating cellular metabolism.

Since 2015, scientists have noticed that circRNAs are enriched in exosomes compared with parental cells, and many investigators have concentrated on the relationship between circRNAs and exosomes [79]. Exosomes also contribute to the mutant metabolism in cancer. They bring the necessary nutrients for tumor growth and transport ncRNAs to regulate the signaling pathways. It is striking that adding exosomes into prostate or pancreatic cancer could promote glycolysis and reduce OXPHOS [80]. However, the underlying mechanism remains to be clarified. According to our collection of metabolic circRNAs, it is possible that exosomes apply circRNAs for the regulation of metabolism.

The metabolic phenotype of cancer stem cells (CSC) has been intensely investigated over the past years. It was reported that high levels of glucose uptake interfered with the natural differentiation of stem cells [81]. And constant stem cell metabolism lays the ground for the Warburg effect. It seems that cellular metabolism controls the “stemness” properties. Publications have reported that circRNAs are implicated with CSCs. For example, circRNA VRK1 was found to be negatively related with breast cancer stem cells [82]. CircBIRC6 could maintain stem cell pluripotency and repress differentiation by binding to miR-34 and miR-145 in human embryonic stem cells (hESCs) [19]. CircBIRC6 also has binding sites for several other miRNAs associated with primary lineage differentiation, including let-7, miR-92, and miR-103. The epithelial-splicing regulatory protein 1 (ESRP1), regulated by pluripotency-associated genes NANOG and OCT4, is responsible for circBIRC6 biosynthesis in hESCs. Therefore, circBIRC6 is probably able to induce the Warburg effect by inhibiting the differentiation of cancer cells. Moreover, FAO plays an indispensable role in CSC growth. Hematopoietic stem cells and leukemia-initiating cells appear to rely on FAO to complete self-renewal [83, 84]. This finding suggests that circRNAs implicating in FAO may regulate CSCs.

One challenge in the study of circRNAs related to cancer metabolism is the lack of direct evidence, and therefore further investigation and validation are warranted in this field. For one thing, although advanced computational models point out several circRNA-miRNA axes, few of them have been verified by experiments in vitro and in vivo. For another, we hypothesize that circRNAs might be involved in cancer metabolism by regulating special TFs directly or indirectly. But the effects of altered TFs might be compensated by corresponding downstream feedback because of complex intracellular regulatory mechanisms. Measurements of metabolic levels before and after circRNA overexpression or knockdown are inevitable. Furthermore, vital oncogenic TFs are involved in more than one metabolic pathway. For example, c-myc can promote both glycolysis and glutamine metabolism. Resultantly, circRNAs may not implicate merely one pathway. Finally, several laboratories have identified that circRNAs play a regulatory role in multiple metabolic diseases, though no sufficient evidence is available to explain their functions in cancer metabolism.

Irrespective of these defects, circRNAs are qualified for a potential therapy to precisely regulate metabolism due to their extremely high stability, strong evolutionary conservation, and unique temporal and spatial expression [77, 85]. With more ncRNAs emerging as important players of metabolism regulation, circRNAs may be able to work as a cut-in point of the whole ncRNA network and bring a promising prospect for the clinical treatment of cancers.

## Conclusion

The purpose of this review is to explore circRNAs that play major roles in cancer metabolism. We have found that several circRNAs are closely associated with carbohydrate, lipid, and amino acid metabolisms. By targeting miRNAs or proteins, circRNAs work as part of the ncRNA regulatory network. In addition, circRNAs may affect exosomes or CSCs to show broad effects. The knowledge about circRNAs may help better understand altered energy metabolism in cancer cells. We envision that with the development of biological research, circRNAs may be utilized for regulating metabolisms in clinical practice in near future.

## Data Availability

Not applicable

## Abbreviations

ceRNA: Competing endogenous RNA
circRNA: Circular RNA
CSC: Cancer stem cell
ESRP1: Epithelial-splicing regulatory protein 1
FA: Fatty acid
FAO: Fatty acid β-oxidation
FAS: Fatty acid synthesis
FASN: Fatty acid synthase
GLUT: Glucose transporter
HCC: Hepatocellular carcinoma
hESCs: Human embryonic stem cells
HK: Hexokinase
HUVEC: Human umbilical vascular endothelial cell
LDHA: Lactate dehydrogenase A
MCT: Monocarboxylate transporter
NADPH: Nicotinamide adenine dinucleotide phosphate
ncRNA: Non-coding RNA
OXPHOS: Oxidative phosphorylation
PCa: Prostate cnacer
PDH: Pyruvate dehydrogenase
PDK: Pyruvate dehydrogenase kinase
PET: Positron emission tomography
PFK: 6-Phosphfructa-1-kinase
PK: Pyruvate kinase
PTBP1: Polypyrimidine tract-binding protein 1
PPAR: Peroxisome proliferator-activated receptor
RBP: RNA-binding protein
Redox: Reduction-oxidation
ROS: Reactive oxygen species
